# Exploring the Potential for Animal Assisted Interventions (AAI) as a Treatment Modality for an Aging Population

**DOI:** 10.1093/geroni/igaf122.3338

**Published:** 2025-12-31

**Authors:** Brigitte Belanger, David Hage

**Affiliations:** Florida Gulf Coast University, Cape Coral, Florida, United States; Florida Gulf Coast University, Fort Myers, Florida, United States

## Abstract

Allied health professionals face significant challenges to providing health care services to aging adults. It is imperative to develop creative strategies to address the complex needs of care recipients and those involved in care provision. Animal Assisted Interventions (AAI) have the potential to positively impact treatment and improve health care outcomes. A recent AAI study with a population experiencing neurocognitive deficits reported benefits for all participants. Six dyads consisting of a care recipient and their care partner attending a SW Florida community dementia support center were invited to participate in 90-minute AAI occupational therapy group sessions over the course of six weeks. Each dyad was placed in one of two experimental groups based on care recipient scores on the Allen Cognitive Level Screen (ACLS-5). Both experimental groups were part of a single-group pre-post-test mixed-method pilot study. As care partners modeled strategies implemented during AAI sessions, such as errorless learning, they began to report improvements in care recipient motivation and activity performance. Additionally, researchers noted increased confidence and patience in caregiving. Researchers hypothesized that AAI could be beneficial to promote health prevention strategies given the interest by participants for future AAI opportunities. The current AAI study explores the effects of human-animal interactions as a strategy to monitor vital signs and assess for early warning signs of atrial fibrillation and atherosclerosis.

